# Activity of Common Thyme (*Thymus vulgaris* L.), Greek Oregano (*Origanum vulgare* L. ssp. *hirtum*), and Common Oregano (*Origanum vulgare* L. ssp. *vulgare*) Essential Oils against Selected Phytopathogens

**DOI:** 10.3390/molecules29194617

**Published:** 2024-09-29

**Authors:** Olga Kosakowska, Zenon Węglarz, Sylwia Styczyńska, Alicja Synowiec, Małgorzata Gniewosz, Katarzyna Bączek

**Affiliations:** 1 Department of Vegetable and Medicinal Plants, Institute of Horticultural Sciences, Warsaw University of Life Sciences—SGGW, Nowoursynowska 166, 02-787 Warsaw, Poland; olga_kosakowska@sggw.edu.pl (O.K.); zenon_weglarz@sggw.edu.pl (Z.W.); sylwia_styczynska@sggw.edu.pl (S.S.); 2 Department of Food Biotechnology and Microbiology, Institute of Food Sciences, Warsaw University of Life Sciences—SGGW, Nowoursynowska 166, 02-787 Warsaw, Poland; alicja_synowiec@sggw.edu.pl (A.S.); malgorzata_gniewosz@sggw.edu.pl (M.G.)

**Keywords:** phytopathogens, essential oils, activity, biopesticides, organic farming

## Abstract

The aim of this study was to determine the activity of common thyme (*Thymus vulgare* L.), Greek oregano (*Origanum vulgare* L. ssp. *hirtum*), and common oregano (*Origanum vulgare* L. ssp. *vulgare*) essential oils (EOs) against selected phytopathogenic microorganisms in relation to their chemical profile. The EOs were obtained from the herbs of 2-year-old plants cultivated in the organic farming system in a temperate climate in Central Europe. The EOs’ composition was determined by GC/MS and GC/FID. The investigated species were represented by the following three chemotypes: ‘thymol’ for common thyme, ‘carvacrol’ for Greek oregano, and mixed ‘caryophyllene oxide + β-caryophyllene’ for common oregano. The antimicrobial activity of the EOs was assessed based on minimum inhibitory concentration (MIC) and minimum bactericidal/fungicidal concentration (MBC/MFC) values. The plant pathogenic bacteria *Pseudomonas syringae*, *Xanthomonas hortorum*, *Erwinia carotovora*, and fungi: *Fusarium culmorum*, *Alternaria alternata*, *Botrytis cinerea*, *Epicoccum purpurascens*, *Cladosporium cladosporioides*, *Phoma strasseri*, and *Pythium debaryanum* were tested. The EOs revealed a stronger inhibitory effect against fungal growth in comparison to bacterial growth (MIC: 0.016–2 µL/mL for fungi and 0.125–4 µL/mL for bacteria). Common thyme and Greek oregano EOs indicated stronger antimicrobial power than common oregano EO. These results were associated with the chemical profile of the analysed EOs. The growth of examined bacteria and fungi strains (in particular, *X. hortorum*, *F. culmorum*, and *P. debaryanum*) were negatively correlated with the content of phenolic monoterpenes and monoterpene hydrocarbons. Among the tested strains, *P. strasseri* turned out to be the most sensitive (MIC 0.016 µL/mL) and *E. carotovora* the most resistant (MIC 0.250–4 µL/mL) to all investigated EOs.

## 1. Introduction

According to the European Green Deal, reducing chemical pesticide use is one of the most important strategic tasks [1]. Recent abuses of synthetic compounds have resulted in numerous harmful effects concerning human health and serious ecological imbalance, followed by the emergence of pathogen resistance. Since chemical plant protection has been recognised as a profit-induced poisoning of the environment, plans to reduce pesticide use are being developed globally. Lately, many countries have reduced their criteria for the minimum residue levels of pesticides in final food products or have even withdrawn particular chemicals from the market. However, in order to feed the ever-growing world population, plant protection is genuinely needed. Thus, the development of low-risk alternative products that are non-toxic to humans, animals, and the environment is of great importance [2,3,4,5,6]. Recently, biopesticides have received great attention as a valuable environmentally friendly replacement for synthetic pesticides. The Environmental Protection Agency defines biopesticides as ‘certain types of pesticides derived from natural sources such as animals, plants, fungi, bacteria and certain minerals’ [5,6]. Among this group, essential oils (EOs) seem to be especially promising since they show a broad spectrum of antimicrobial activity, as well as indicate insecticidal, anti-feedant, repellent, growth regulator, and anti-vector properties [7,8,9,10]. Many EOs are used as flavouring agents in foodstuffs and beverages. They are labelled ‘generally recognized as safe’ (GRAS) by the US Food and Drug Administration. This special regulatory status, ensuring safety for non-target organisms and the environment, would have made possible the fast-track registration and commercialization of EO-based pesticides [2,6].

Among plant pathogens, fungi (mainly *Alternaria* spp., *Botrytis* spp. And *Fusarium* spp.) are responsible for nearly 30% of all crop diseases. They cause quality losses worth billions of dollars worldwide each year. Phytopathogenic bacteria, e.g., *Erwinia* spp., *Pseudomononas* spp., and *Xanthomonas* spp. strains, also have a considerable negative economic impact on food production [3,11,12,13]. Both fungi and bacteria are responsible for various complex plant diseases, especially rots and/or wilts appearing in the juvenile stage of plant development, then moulds, blights, and spot diseases usually present when plants are mature. Altogether, this may cause not only serious losses in plant productivity, followed by a low yield, but also significant decreases in the quality of the raw materials obtained. The most dangerous threat seems to be contamination by the fungal secondary metabolites—mycotoxins. Even trace amounts of these substances in plant products may lead to harmful impacts on human health, including mutagenic, teratogenic, and hepatotoxic effects [14,15]. The problem of mycotoxin residues is of great concern, especially in organic farming production, where chemical plant protection is banned. Therefore, EO-based pesticides with well-proven antifungal and antibacterial activities appear to be a promising alternative. These activities, already summarised by Raveau et al. [3], Chang et al. [6], and Sighn et al. [15], include the loss of integrity of microbe cell membranes, leakage of electrolytes, loss of intracellular constituents, protein disruption, and, finally, the inhibition of the respiratory metabolism of bacteria and/or fungi.

Nowadays, plants belonging to the Rutaceae and Mirtaceae family, such as orange (*Cirus* x *sinensis*) or cloves (*Syzygium aromaticum*), seem to be the most popular worldwide as a source of EOs used against phytopathogens and are officially classified by the EU as products permitted for application in organic farming [16]. However, their price is high, and the quality often does not meet acceptable standards. Moreover, in the case of Central Europe, the raw materials (e.g., oranges) and/or EOs alone need to be transported from warmer climates. This generates a carbon footprint. Taking into account European Green Deal assumptions, including the closed-loop economy idea, locally cultivated plants should be prioritised over exotic ones [1]. They would provide cheaper, easy-to-obtain, and commercially available raw materials. Regarding this, growing attention has recently been paid to species from the Lamiaceae family, e.g., *Thymus* spp., *Origanum* spp., *Salvia* spp., and *Satureja* spp., etc. Until now, these species have been mainly used for herbal drugs and spices. Compounds that dominate in their EOs, mainly phenolic monoterpenes (thymol, carvacrol) and oxygenated monoterpenes (e.g., thujone), reveal a wide spectrum of antimicrobial activity [17]. Although substantial literature data are available on this issue, some problems are apparent. The results obtained are often not comparable due to different methods of EO application, in addition to the confounding influence of various strains of target microorganisms.

Some studies seem to be incomplete since the antimicrobial activity of EOs is usually determined separately from their chemical composition. This is especially important in the case of the Lamiaceae species (especially *Origanum* genus) due to their huge chemical polymorphism reflected in the creation of different chemotypes [18,19,20]. Moreover, raw materials providing EOs intended to be applied as biopesticides should be free of contamination, especially when given pesticide residues. Therefore, it is crucial to cultivate EOs-bearing plants in the organic farming system. 

The purpose of the present study was to determine the activity of EOs derived from the following three Lamiaceae species and subspecies: common thyme (*Thymus vulgare* L.), Greek oregano (*Origanum vulgare* L. ssp. *hirtum*), and common oregano (*Origanum vulgare* L. ssp. *vulgare*) cultivated in the organic farming system in the temperate climate of Central Europe, against selected phytopathogenic fungi and bacteria, in relation to their chemical profile. 

## 2. Results

In the case of common thyme, the mass of the herb was at the level of 175.8 g FW and 58.7 g DW per plant. These parameters were visibly higher for Greek oregano (482.1 g FW; 226.2 g DW per plant) and common oregano (534.3 g FW; 190.7 g DW per plant). The highest EO content was noticed in the Greek oregano herb (3.2 g/100 g), followed by common thyme (1.7 g/100 g) and common oregano (0.6 g/100 g) (Table 1).

**Figure 1:**
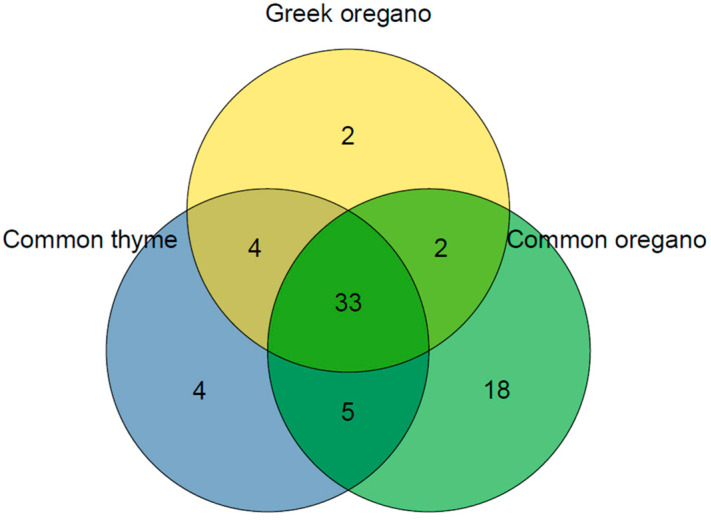
Venn diagram based on the percentage share of EO compounds.

**Figure 2:**
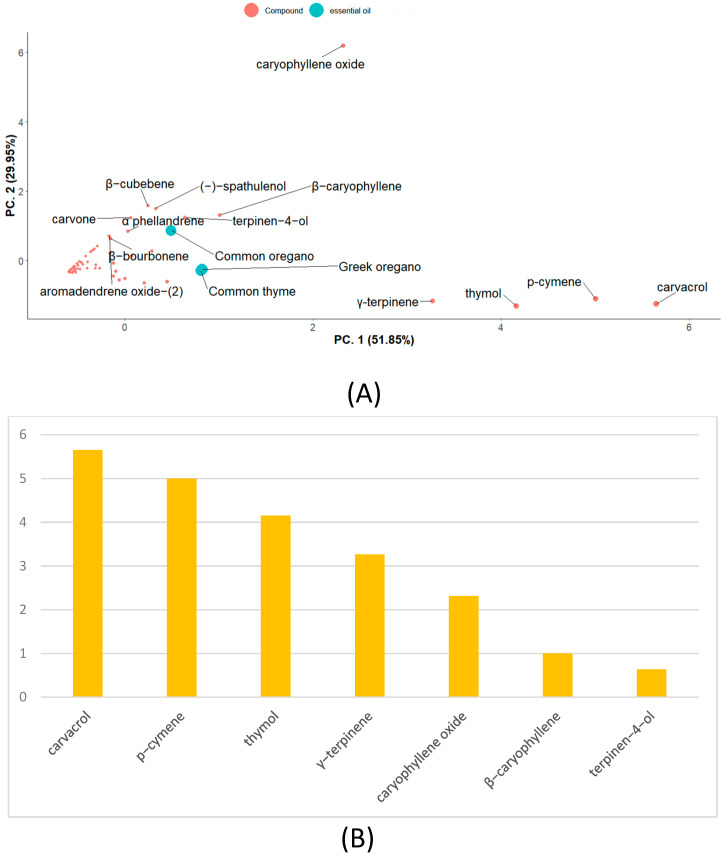
PCA map (**A**) and VIP values (**B**) from PCA analysis based on the chemical profiles of EOs.

Sixty-eight compounds were identified among the EOs investigated, comprising 93.24–98.23% of the total fractions. Here, 46 compounds were identified in common thyme EO, 41 in Greek oregano EO, and 58 in common oregano EO (Figure 1). In common thyme and Greek oregano EOs, monoterpenes build the fundamental part. This group consisted of the following three subgroups: phenolic monoterpenes with the domination of thymol (up to 27.28% for common thyme) and carvacrol (up to 35.79% for Greek oregano); monoterpene hydrocarbons with γ-terpinene and *p*-cymene present in the highest amounts (11.67 and 18.20% for common thyme; 13.76 and 17.01% for Greek oregano, respectively); and oxygenated monoterpenes (up to 12.99% for common thyme and 6.65% for Greek oregano, respectively). In turn, common oregano EO was characterized by the domination of oxygenated sesquiterpenes (up to 35.80%), represented mainly by caryophyllene oxide (18.89%). Sesquiterpene hydrocarbons (e.g., β-caryophyllene, β-bourbonene, β-cubebene), followed by oxygenated monoterpenes (e.g., terpinen-4-ol, carvone, linalool), were present here in the considerable amounts, too (Table 2). In general, the dominant constituents, namely, carvacrol, *p*-cymene, thymol, and γ-terpinene, followed by caryophyllene oxide, β-caryophyllene, and terpinen-4-ol, differentiated the investigated EOs the most (Figure 2).

In this study, MIC and MBC/MFC values of the analysed EOs express the strength of their action against pathogenic microorganisms. The EOs revealed a weaker inhibitory effect (MIC) on bacterial growth than on fungal growth. The inhibitory effect MIC ranged from 0.125 to 4 µL/mL (for bacteria) and from 0.016 to 2 µL/mL (for fungi) (Table 3). A much stronger antibacterial activity of common thyme and Greek oregano EOs was observed compared to that of common oregano EO. The inhibitory effect of EOs varied depending on the strain. Of the tested microorganisms, the fungus *Phoma strasseri* turned out to be the most sensitive to all three EOs (MIC 0.016 µL/mL), and the most resistant was the bacterium *Erwinia carotovora* (MIC 0.250–4 µL/mL). 

The bactericidal and fungicidal effects of the analysed EOs varied distinctly. The MBC values of all three EOs ranged between 0.125 and 8 µL/mL, whereas the MFC ranged between 0.032 and 8 µL/mL (Table 3). Thyme and Greek oregano EOs revealed bactericidal and fungicidal effects in similar MBC and MFC ranges, whereas common oregano EO showed much weaker bactericidal and fungicidal effects.

A varied spectrum of EO action was found depending on the concentration used (Table 4). All the microorganisms were inhibited by thyme and Greek oregano EOs at a concentration of 0.25 µL/mL. The same concentration of common oregano EO inhibited only 30% of the strains. It showed 100% activity only at a concentration of 4 µL/mL.

**Figure 3:**
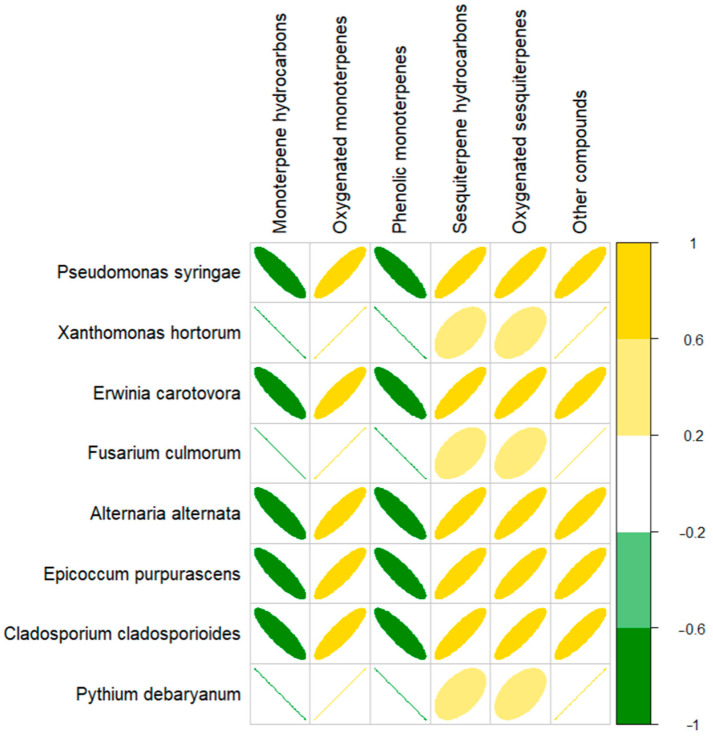
Spearman rank correlation plot based on EO chemical profiling.

Figure 3 shows the Spearman rank correlation between antimicrobial activity and the EO chemical profile. It was observed that the growth of the examined strains (in particular, *Xanthomonas hortorum*, *Fusarium culmorum*, and *Phytium debaryanum*) was negatively correlated with the content of phenolic monoterpenes and monoterpenes hydrocarbons in the investigated EOs.

## 3. Discussion

Results concerning the weight of the herbs of the investigated species/subspecies indicate that they create satisfying yields when grown in the climatic conditions of Central Europe in the organic farming system. It is especially meaningful in the case of common thyme and Greek oregano, both of Mediterranean origin. These plants appeared to be well adapted to the temperate zone climate, and, importantly, still kept their typical characteristics, such as a high EO content. It should be underlined that the mass of the Greek oregano herb was comparable to that of the common oregano herb, the subspecies native to Central Europe. Overall, the cultivation parameters of the species/subspecies investigated in this work, their morphological and developmental traits, as well as the yield of raw materials and their EO content, have been briefly discussed in our previous articles [21,22,23,24,25]. 

Regarding the polymorphic nature of aromatic plants, the composition of EOs seems to be a crucial parameter of their quality since this substance strongly affects the biological activity of raw materials [26]. In our work, based on the dominant compounds in the EOs, it was possible to classify them as particular chemotypes, characteristic of each species/subspecies. When given common thyme, the predominance of thymol led to qualifying the examined EO as a ‘phenolic/thymol’ chemotype. So far, six main chemotypes were distinguished within common thyme, as follows: phenolic (thymol, carvacrol) and non-phenolic (linalool, geraniol, α-terpineol, trans-thujan-4-ol/terpinen-4-ol) [27,28,29,30,31]. In the case of Greek oregano and common oregano, the chemical composition of the EOs allowed their classification as ‘phenolic/carvacrol’ and ‘sesquiterpenes/caryophyllene oxide + β-caryophyllene’ chemotypes, respectively. These results correspond well with the literature data. Among six subspecies of *O. vulgare*, the southernmost ones, such as *O. vulgare* ssp. *hirtum* (Greek oregano), are rich in EO with the frequent occurrence of the pure phenolic (carvacrol or thymol) chemotype [32,33,34]. In turn, *O. vulgare* ssp. *vulgare* (common oregano), similar to other subspecies originating from the central part of Europe, is regarded as a rather poor source of EO, characterized by the domination of the following non-phenolic compounds: sabinyl (i.a. sabinene, *cis/trans* sabinene hydrate and its acetates); acyclic (i.a. β-ocimene, β-myrcen, linalyl acetate, linalool) and/or sesquiterpenes (i.a. germacrene D, β-caryophyllene and caryophyllene oxide) [34,35,36,37]. Nevertheless, irrespective of the species and subspecies, carvacrol and/or thymol chemotypes are considered to be the most valuable from an industrial perspective due to the proven biological activity of both phenolic monoterpenes [38]. 

Due to the recent EU regulations on the sustainable use of plant protection products, a 50% reduction of chemical pesticides is a target to achieve by the end of 2030 [39]. So far, the usage of certified seeds, followed by proper soil management, including crop rotation, is visibly insufficient to control plant diseases. EOs are regarded as new, powerful ‘green’ products to counteract the effect of phytopathogenic microbes [3,6]. 

About 350 species, subspecies, and pathovars of bacteria belonging to phyla Protecobacteria, Actinobacteria, and Firmicutes are known to be phytopatogenic [11]. Among them, one of the best known—even used as a model organism to understand the biology of plant disease—is *Pseudomonas syringae*. This Gram-negative bacterium causes dangerous diseases in various crops, including vegetables, fruits, and cereals. The symptoms manifest, for example, as a canker of stone fruit trees and/or spot-leaf disease. Pathogenic and non-pathogenic isolates of *P. syringae* are also commonly found as epiphytes in the environment. Being easily transmitted by wind and rain, it easily spreads worldwide [40,41]. In our work, in order to express the antibacterial activity of the EOs investigated against target bacteria, MIC and MBC were determined. Results showed that both thyme and Greek oregano EOs effectively inhibited the growth of *P. syringae,* while the bactericidal power towards this strain was higher in the case of thyme EO (Table 3 and Table 4). The effectiveness of these EOs against *P. syringae* was observed earlier by other authors [42,43,44,45,46]. According to Kokoskova et al. [42], both thyme and oregano EOs revealed stronger antibacterial activity when compared to lemon balm and mint EOs. Oliva et al. [43] claim that oregano EO is more effective than streptomycin. 

*Erwinia carotovora* (syn. *Pectobacterium carovotorum*) is an important pathogenic bacterium responsible for a soft-rot disease, which is dangerous, especially in the production and storage of onion and potato. The bacterium, generally known as a tissue maceration agent, is able to penetrate the host plant due to the activity of specific cell wall-degrading enzymes such as pectate lysae, polygalacturonase, protease, and cellulase [47]. Results obtained in the present study indicate that *E. carotovora* was more susceptible to common thyme and Greek oregano EOs in comparison with common oregano EO. It is worth noting that both EOs revealed similar levels of bacteriostatic and bactericidal activity (Table 3). Zhang et al. [48] noticed the promising potential of oregano and cloves EOs in preventing the store soft-rot decay of onion. It was observed that the EOs of other Lamiaceae plants, which include hyssop, savory, and mint, may be an efficient agent for controlling postharvest diseases caused by *E. carotovora* [47,49]. 

*Xanthomonas* is a wide genus that includes various bacteria species, subspecies, and pathovars. Among them, *Xanthomonas hortorum* is a common reason for the bacterial blight of geranium, a disease leading to serious problems in the production of this ornamental plant [14]. In our work, we noticed that, similar to the bacteria described above (*Pseudomonas syringae* and *Erwinia carotovora*), *X. hortorum* was also more susceptible to common thyme and Greek oregano EOs than common oregano EO (Table 3). According to the results of other authors, *Xanthomonas* strains appeared to be sensitive to different EOs, e.g., oregano, marjoram, savory, tansy, and cloves [45,50,51,52]. 

The observed antibacterial activity of EOs may be related to their chemical composition, especially with the domination of phenolic monoterpenes in Greek oregano and common thyme. Here, carvacrol and thymol are known for their antibacterial activity against a broad range of both Gram-positive and Gram-negative bacteria. Strains used in our work belong to Gram-negative bacteria, and all three were susceptible to EOs rich in these compounds (thymol in common thyme and carvacrol in Greek oregano). In turn, common oregano EO, characterized by a high share of oxygenated sesquiterpenes (with caryophyllene oxide as a dominant) followed by a slight content of phenolic monoterpenes, was found not to be as effective towards the investigated bacteria (MIC value 2–4). Both phenolic monoterpenes, due to their lipophilic character, are able to penetrate not only the cell wall and cell membrane but also the outer layer of Gram-negative bacteria. In general, EOs’ antibacterial mechanisms manifest mainly in the loss of integrity of the cell walls and membranes, leakage of electrolytes, protein disruption, loss of intracellular constituents, increase of electrical conductivity, and the inhibition of respiratory metabolism followed by the breakdown of growth and overall developmental processes [3,6,15]. With respect to antifungal activity, the same mode of action of EOs was observed. Moreover, EOs disrupt fungal cells via the inhibition of ergosterol biosynthesis [53,54]. Ergosterol, as the main sterol present in fungal cell membranes, maintains the fluidity and integrity of the fungal cell. EOs can also induce changes in mitochondrial membrane potential, extracellular matrix acidification, alteration in activities of mitochondrial ATPase and dehydrogenase enzymes, and increased endogenous reactive oxygen species production [54]. Microscopic studies confirmed that EOs could destroy the ultrastructure of hyphae and conidia [6].

Over 19,000 phytopathogenic fungi are known to cause plant diseases [13]. Among them, *Botrytis cinerea*, a common reason for grey moulds, is regarded as one of the most dangerous for the agricultural sector in temperate climates [14,55,56]. The results of our work showed that all tested EOs effectively inhibited the growth of this species, with the minimum inhibitory concentration (MIC) at the level of 0.062 µL/mL. However, as regards the fungicidal activity, common thyme EO appeared to be most effective (MFC 0.250 µL/mL) in comparison with Greek oregano (MFC 500 µL/mL) and common oregano (MFC 8 µL/mL) (Table 3 and Table 4). According to Zhao et al. [57], both oregano EO and pure phenolic monoterpenes exhibited strong antifungal activity against *B. cinerea*: thymol had the lowest EC50 against mycelial growth, followed by carvacrol and EO. The results of Ben Ghnaya et al. [58] indicate the effectiveness of eucalyptus EO towards *B. cinerea*, while Montenegro et al. [59] listed *Mentha pulegium* EO.

*Alternaria alternata* is one of the most common saprophytes found throughout the world. It causes a variety of diseases, including dark leaf spots, brown spots, black rot, etc. [60]. Being the major postharvest fungal pathogen, *A. alternata* is responsible for many negative symptoms, such as the softening of fruits (especially grapes and citrus), followed by the loss of their weight and colour changes [61]. It also causes the dark leaf spot disease, commonly occurring on plantations of vegetables from the Brassicaceae family, as well as the black point disease of cereals [14]. In our work, *A. alternata* was noticed to be sensitive to thyme and Greek oregano EOs, with their fungistatic activity (expressed as MIC value) at the level of 0.062 µL/mL, while common oregano inhibited the growth of this fungi when the MIC value reached 1 µL/mL. With respect to fungicidal activity, Greek oregano EO was the most effective (MFC 0.062 µL/mL) (Table 3 and Table 4). Ghuffar et al. [61] investigated the antifungal activity of thyme, fennel, garlic, ginger, and caraway EOs against *A. alternata*, and observed that common thyme EO was more effective than the other EOs. Similar results were obtained by Aslam et al. [62] and Perina et al. [63]. In turn, Affes et al. [64] indicate that the combination of laurel and peppermint EOs is the most preferable for *A. alternata* treatment. 

Similar to *Alternaria*, members of *Cladosporium* are widely distributed around the world. They occur mainly on decayed organic products but generally can be found in both the soil and air. Despite being saprophytic, *Cladosporium* ssp. may cause plant diseases such as the black point of cereals, the leaf spots in pecan trees and sunflowers, the scab in papaya, and the rot in grapes [65]. It can also lead to human disorders and is considered an important food contaminant and a serious respiratory and skin allergen [66]. The results of the present study have shown that the growth of *Cladosporium cladosporioides* can be successfully limited by common thyme and Greek oregano EOs (MIC = 0.016 µL/mL), followed by common oregano EO (MIC 0.125 µL/mL). However, fungicidal activity appeared to be effective only in the case of thyme EO (Table 3).

Along with other phytopathogens, especially *Alternaria* and *Cladosporium*, *Epicoccum* may also cause black point disease of cereals (wheat, barley, and rye) [14]. In our work, we have noticed that the growth of *Epicoccum purpurascence* can be effectively inhibited by both common thyme and Greek oregano EOs (MIC 0.016 µL/mL), while the activity of common oregano EO was visibly lower (MIC 2 µL/mL). In turn, the fungicidal power was the highest in the case of Greek oregano EO (Table 3). Results given by Gleń-Karolczyk and Boligłowa [67] showed that the juniper EO revealed strong fungicidal activity against *E. purpurascence.*

Among phytopathogenic fungi, the species belonging to *Fusarium* and *Aspergillus* not only decrease the yield and quality of plant products but are also considered human health risk agents since they produce mycotoxins [14]. Within this group, in the present study, we examined *Fusarium culmonorum,* which mainly infects cereals at different stages of their development and causes brown foot rot and/or fusarium wilt symptoms. The species synthesize mycotoxins such as nivalenol and deoksynivalenol (DON), which may contaminate food and lead to serious health problems including immune system suspension [68]. Thus, the level of DON in food products is regulated by the EU [69]. It is worth noting that the production of mycotoxin in fungal cells may be limited by EO components via the inhibition of methylglyoxal biosynthesis, which is produced by fungi to enhance the production of mycotoxins, especially aflatoxin [70]. Of the EOs tested in our work, those rich in phenolic monoterpenes (common thyme and Greek oregano EOs) were characterized by stronger antifungal activity than common oregano EO (Table 2 and Table 3). Other authors listed eucalyptus and cloves EOs as effectively inhibiting the growth and development of the pathogen [58,71]. 

*Phoma strasseri* (syn. *Boeremia strasseri*) causes black stem and rhizome rot, also called phomosis, which is one of the most severe diseases of peppermint, followed by other Lamiaceae species [72,73,74]. The disease symptoms are visible on the stems, first in the form of necrotic, slightly hollow spots enfolding the stem up to around 10 cm from the base. With time, the tissue in the location of the spots rots. The yield losses resulting from peppermint infection can even reach 90% [75]. The results obtained in our studies showed that *P. strasseri* is visibly susceptible to all tested EOs, with their fungistatic activity at the level of 0.016 µL/mL. Regarding fungicidal activity, Greek oregano was found to be the most effective (MFC 0.032 µL/mL), followed by thyme (MFC 0.064 µL/mL) and common oregano (MFC 0.25 µL/mL) (Table 3). It should be emphasised that among the phytopathogens investigated, *P. strasseri* was the most sensitive to all EOs applied.

The *Pythium* genus is the most destructive phytopathogenic agent for all types of cultivated plants. It is associated with seedling tipping, also called damping off and root rot. The root rot affects the plant during germination, attacking the organs of the seedling and causing it to tip over. The symptoms of damping off begin from rotting in the basal region of the stem, and it can start even before the pathogen penetrates the somatic structures of the tissue through the action of enzymes. The disease occurs widely in the temperate climate zone, especially in the case of plants sown directly into the field in the spring [76]. The species tested in the present work, *Pythium debargyanum,* was the most sensitive to Greek oregano EO (MIC = 0.016 µL/mL, MFC = 0.032 µL/mL), in comparison with common thyme and common oregano. Here, the antifungal activity of common oregano EO (expressed as MIC and MFC values) was at a level of 1 µL/mL, which indicates the same fungistatic and fungicidal power of the EO (Table 3). According to Siddiqui et al. [77], the EO of *Mikania scandes* effectively limited the mycelial growth of *Phytium graminicola*. It is worth noting that the chemical composition of both *M. scandes* and common oregano EOs is characterized by a high share of sesquiterpenes, with the domination of caryophyllene and caryophyllene oxide [77]. 

To sum up, the antimicrobial properties of thyme and Greek oregano seem to be similar, unlike common oregano, which indicated much weaker activity. This may be related to the chemical composition of the compounds present in their EOs. Compositionally, thyme and Greek oregano EO are very similar (Figure 2). The main compounds (thymol, carvacrol, γ-terpinene, p-cymene) reveal strong antibacterial and antifungal properties. According to literature data, there are several mechanisms responsible for the activity of this group of compounds. One of them is the disruption of the metabolism within the cell. These compounds are able to damage cell membranes and react with active sites of enzymes [78]. Moreover, the destruction of cell membranes by permeabilization and depolarization of the cytoplasmic membrane, followed by the loss of cell wall strength of fungi, was reported [79]. Studies by Oliveira et al. (2020) showed the inhibitory effect of thyme oil rich in thymol and carvacrol on the expression of genes such as the global regulator gene of fungal secondary metabolism and two genes responsible for virulence mechanisms [80]. Also, the presence of compounds that are precursors of phenolic compounds, such as γ-terpinene and p-cymene, has demonstrated properties against plant pathogens [81]. The demonstration of a negative correlation between monoterpene hydrocarbons or phenolic monoterpenes and the microorganisms studied seems to be an important aspect of our work. There was no significant negative correlation between oxygenated sesquiterpenes and sesquiterpenes hydrocarbons, dominant in common oregano EO. Previous studies confirm that EOs rich in sesquiterpenes showed much weaker antimicrobial properties than those containing thymol and carvacrol [81]. Research conducted on fungi has shown that the MIC values for terpinen-4-ol range from 20 to 1420 µg/mL [82]. The mechanism of action of terpene-4-ol is based on inhibiting growth through cell membrane disruption, reducing antioxidant enzyme activities (catalase, peroxidase, superoxide dismutase) and interrupting the tricarboxylic acid cycle [83].

In general, the results obtained in our study show that both the antibacterial and antifungal activity of the EOs tested is visibly associated with their chemical composition, especially with the domination of phenolic monoterpenes: carvacrol in Greek oregano and thymol in common thyme. It was observed that, despite belonging to the same species, Greek oregano and common oregano differ significantly in terms of EO composition and biological activity, which was also reported in our previous articles [21,22,23,24]. Thus, in the case of the *Origanum* plants, the potential application of their EOs as biopesticides in organic agriculture must be preceded by subspecies identification followed by recognition of their chemotypes. Regarding the promising antimicrobial activity of Greek oregano and common thyme EOs against the above-mentioned phytopathogens, it may be suspected that these plants will soon become a serious source of EO-based biopesticides. However, despite the proven efficacy of EOs, they are still not in widespread application due to their high volatility, low stability and water solubility, composition variability, sensitivity to light and temperature, and phytotoxic effects. In order to achieve the necessary persistence and efficacy of EOs, a proper formulation is required, including encapsulation and/or emulsification [7]. Due to the above-mentioned limitations, most of the studies on EOs are limited to laboratory conditions at the research but not the application stage; farmers still lack satisfactory practical alternatives to synthetic pesticides [5]. Therefore, future research concerning the in vivo application of EOs should be undertaken.

## 4. Materials and Methods

### 4.1. Plant Material

The objects of the study were common thyme (*Thymus vulgaris* L.; ‘Standard Winter’; seeds purchased at Jelitto^®^, Schwarmstedt, Germany), Greek oregano (*O. vulgare* subsp. *hirtum*; seeds purchased at Jelitto^®^, Schwarmstedt, Germany) and common oregano (*O. vulgare* subsp. *Vulgare*; seeds purchased at FZL Sp. z o.o., Kruszynek, Poland) cultivated on the experimental field of the Department of Vegetable and Medicinal Plants, Warsaw University of Life Sciences (WULS-SGGW) (5210180 N; 2105234 E), in alluvial soil. The seedlings of these plants were produced in a greenhouse. Six to eight weeks after sowing, the seedlings were transplanted into multi-pots and then, after the next six weeks, they were used to establish the field experiment (mid-May 2017). The randomized block design (60 seedlings per plot; in 3 replications) was applied, with a spacing of 40 × 60 cm for Greek oregano and common oregano and 30 × 50 cm for common thyme. The herbs (upper, not woody parts of shoots) of these plants were harvested in June 2018 from 2-year-old plants at the beginning of their blooming. The fresh and dry weights of the herbs were determined (g per plant). After drying at 35 °C, the herb was powdered and prepared for chemical analysis. The plant production was certified in accordance with organic production rules by Ekogwarancja Ltd. (Warsaw, Poland). Climatic and soil parameters were recorded (Table 5 and Table 6).

### 4.2. EO Extraction and GC-MS/GC-FID Analysis

Sixty grams of air-dried raw material was subject to hydrodistillation for 3 h using a Clevenger-type apparatus. The EO content was expressed as g per 100 g of dry weight of raw material. The EOs were stored in dark vials at 4 °C. The analysis was performed using an Agilent Technologies 7890A (Agilent, Santa Clara, CA, USA) gas chromatograph combined with a flame ionization detector (FID) and MS Agilent Technologies 5975C Inert XL_MSD with Triple Axis Detector (Agilent Technologies, Wilmington, DE, USA). A polar capillary Omegawax^®^ column (30 m × 0.25 mm × 0.25 µm film thickness) was applied. Separation conditions were as follows: oven temperature isotherm at 60 °C for 2 min, then it was programmed from 60 °C to 220 °C at a rate of 4 °C per minute and held isothermal at 220 °C for 5 min. Separation conditions were previously described by Bączek et al. [84]. The EO compounds identification was based on the comparison of mass spectra from the Mass Spectral Database, as follows: NIST08, NIST27, NIST147, NIST11, Wiley7N2, and on a comparison of retention indices (RI) relative to the retention times of a series of n-hydrocarbons (C7–C30) with those reported in the literature.

### 4.3. Antimicrobial Activity

#### 4.3.1. Target Microorganisms

The reference strains originated from the Bank of Pathogens of the Institute of Plant Protection—National Research Institute (IPP—NRI) in Poznań, Poland. The study used three bacterial strains: *Pseudomonas syringae* IOR 2146, *Xanthomonas hortorum* IOR 1832, and *Erwinia corotovora* IOR 1826, and various fungi were also incorporated, including *Fusarium culmorum* IOR 2218, *Alternaria alternata* IOR 2044, *Botrytis cinerea* IOR 2193, *Epicoccum purpurascens* IOR 1741, *Cladosporium cladosporioides* IOR 2017, *Phoma strasseri* IOR 1801, and *Pythium debaryanum* IOR 2164.

##### Minimum Inhibitory Concentration (MIC) and Minimum Bactericidal/Fungicidal Concentration (MBC/MFC)

In order to determine the MIC of the EOs, a serial microdilution method was used, according to the Clinical Laboratory Standards Institute standards for bacteria and EUCAST (2022). Two series of dilutions of the examined EOs were prepared in Müller–Hinton broth (MHB, Merck, Darmstadt, Germany) for bacteria and RPMI 1640 medium (Merck, Darmstadt, Germany) with 2% glucose in MOPS buffer (Merck, Darmstadt, Germany) for fungi in the concentration range of 0.016–64 µL/mL using 96-well plates. An inoculum of microorganisms was added to each well of the plate containing 250 μL of medium (MBH or RPMI 1640) so that the final number was 5 × 10^5^ cfu/mL. The plates with bacteria were incubated at 37 °C for 20 h, and plates with fungi were incubated at 28 °C for 48 h (*B. cinerea* mould was incubated at 20 °C). After the incubation time, 25 μL of resazurin at a concentration of 0.02% *m*/*v* (filtered through a 0.22 μm sterile filter) was added to each well. The plates were then incubated at an appropriate temperature for 2 h. The growth of the microorganisms was assessed based on the change in resazurin colour from violet to pink. The MIC value was defined as the lowest concentration of essential oil in which no visible growth of microorganisms was observed (no change in resazurin colour). The medium with the inoculum was used as a positive control. 

The MBC/MFC assay was performed after an MIC test was completed. The test consisted of inoculating 0.1 mL cultures from all wells in which no bacterial or fungal growth had been observed (after the MIC test) on Müller–Hinton Agar (MHA, Merck, Darmstadt, Germany) for bacteria or Sabouraud Dextrose Agar (SDA, Merck, Darmstadt, Germany) for fungi. Incubation of bacteria was carried out at 37 °C for 24 h, and in the case of fungi, at 28 °C for 48 h (*B. cinerea* was incubated at 20 °C). Then, the number of colonies grown was calculated. MBC/MFC is defined as the lowest concentration of essential oil that results in a 99.9% reduction in the number of viable microorganisms.

The percentage of the activity spectrum of the EOs was determined based on the MIC (A) value according to the following formula:

A (%) = [(number of tested strains inhibited by the essential oil)/(total number of tested strains)] × 100

Percentage activity indicates the overall antimicrobial potency of the EOs, meaning the percentage of bacteria and fungi that were inhibited by the EOs.

### 4.4. Statistical Analysis

Statistical calculations for the Spearman correlation rank and principal component analysis (PCA) were carried out using the STATISTICA 13 PL (StatSoft, Cracow, Poland) computer program. Spearman’s rank correlation plots, Venn diagrams, and principal component analysis (PCA) plots were obtained using the R platform.

## Figures and Tables

**Table 1:** Mass of raw materials (g/plant) and EO content (g/100 g DW).

	Common Thyme	Greek Oregano	Common Oregano
Fresh mass of herb	175.8 ± 40.6	482.1 ± 117.0	534.3 ± 110.2
Dry weight of herb	58.7 ± 14.5	226.2 ± 58.8	190.7 ± 55.1
Essential oil content	1.7 ± 0.1	3.2 ± 0.5	0.6 ± 0.01

**Table 2:** The composition (% peak area) of the EO samples.

No.	Compound	RI ^1^	Common Thyme	Greek Oregano	Common Oregano
1	α-pinene	1029	2.33	3.39	0.08
2	camphene	1074	0.79	0.68	0.56
3	β-pinene	1112	0.38	4.08	0.13
4	3-carene	1147	0.16	0.27	0.12
5	α-terpinene	1185	2.70	4.48	0.39
6	D-limonene	1204	2.26	0.58	0.59
7	a phellandrene	1212	0.70	0.04	3.53
8	1.8-cineole	1214	1.18	0.00	1.79
9	(*E*)-2-hexenal	1216	0.00	0.06	0.03
10	*trans* β-ocimene	1236	0.04	0.14	0.57
11	γ-terpinene	1250	11.67	13.76	1.98
12	*p*-cymene	1275	18.20	17.01	4.02
13	*m*-cymene	1281	0.00	0.00	0.47
14	terpinolene	1284	0.17	0.46	0.30
15	anisole	1357	1.56	1.30	1.82
16	3-octanol	1391	0.16	0.04	0.27
17	α-tujone	1425	0.00	0.00	0.11
18	p-cymenene	1440	0.08	0.00	0.00
19	β-tujone	1448	0.00	0.40	0.13
20	1-octen-3-ol	1446	1.17	1.06	1.12
21	linalool oxide	1452	0.03	0.00	0.00
22	*trans*-2-caren-4-ol	1460	0.05	0.00	0.00
23	*trans*-p-menthone	1464	0.00	0.00	0.13
24	camphore	1518	0.48	0.00	0.05
25	β-bourbonene	1530	0.05	0.20	2.80
26	β-cubebene	1539	0.05	0.16	5.51
27	linalool	1542	3.22	0.26	2.44
28	bornyl acetate	1575	0.19	0.11	0.05
29	β-copaene	1581	0.04	0.06	0.00
30	*trans*-p-mentha-2-en-1-ol	1583	0.08	0.12	0.79
31	β-caryophyllene	1594	2.20	3.34	5.64
32	terpinen-4-ol	1597	1.12	2.45	5.08
33	sabina ketone	1610	0.00	0.00	0.84
34	*cis*-p-mentha-2-en-1-ol	1618	0.05	0.06	0.35
35	*cis*-terpineol	1621	1.08	0.64	0.60
36	dihydrocarvone	1630	0.04	0.15	0.00
37	γ-elemene	1641	0.00	0.08	0.00
38	α-humulene	1658	0.07	0.38	0.67
39	viridiflorene	1670	0.00	0.07	0.00
40	*trans*-terpineol	1674	0.39	0.60	0.45
41	borneol	1684	2.08	1.73	0.00
42	p-menth-6-en-2-one	-	2.77	0.13	0.25
43	*trans*-piperitol	1725	0.00	0.00	0.13
44	carvone	1740	0.00	0.00	4.47
45	β-bisabolene	1743	0.14	1.25	0.76
46	α-farnesen	1749	0.00	0.00	1.89
47	δ-cadinene	1760	0.29	0.26	0.91
48	geranyl acetate	1763	0.00	0.00	0.16
49	α-cadinene	1770	0.24	0.08	0.13
50	cuminal	1786	0.00	0.00	0.39
51	*trans*-calamene	1827	0.00	0.00	0.46
52	geraniol	1845	0.19	0.00	0.00
53	p-cymenol	1851	0.04	0.00	0.20
54	thymol acetate	1896	0.07	0.27	0.00
55	caryophyllene oxide	1976	0.43	0.35	18.89
56	germacrene-D-4-ol	2027	0.00	0.00	0.36
57	humulene epoxide II	2041	0.00	0.00	2.12
58	cubenol	2073	0.04	0.00	0.22
59	*trans*-longipinocarveol	2091	0.00	0.00	1.63
60	globulol	2097	0.00	0.00	0.67
61	(−)-spathulenol	2125	0.05	0.95	5.38
62	tau-cadinol	2141	0.32	0.00	0.32
63	thymol	2166	27.28	0.99	2.91
64	α-muurolol	2193	0.00	0.00	0.24
65	carvacrol	2213	6.61	35.79	3.97
66	α-cadinol	2215	0.00	0.00	1.75
67	aromadendrene oxide-(2)	2232	0.00	0.00	2.95
68	eudesma-7.11-dien-4-ol	2254	0.00	0.00	1.27
	Total identified		93.24	98.23	95.84
	Monoterpene hydrocarbons		39.48	44.89	12.74
	Oxygenated monoterpenes		12.99	6.65	18.41
	Phenolic monoterpenes		33.96	37.05	6.88
	Sesquiterpene hydrocarbons		3.08	5.88	18.77
	Oxygenated sesquiterpenes		0.84	1.30	35.80
	Other compounds		2.89	2.46	3.24

RI ^1^—experimental retention index on polar column.

**Table 3:** MIC (MBC/MFC) values of EOs (µL/mL).

Species	Common Thyme	Greek Oregano	Common Oregano
Bacteria			
*Pseudomonas syringae*	0.125 (0.125)	0.125 (0.250)	4 (4)
*Xanthomonas hortorum*	0.250 (0.250)	0.125 (0.250)	2 (2)
*Erwinia carotovora*	0.250 (0.250)	0.250 (0.250)	4 (8)
Fungi			
*Fusarium culmorum*	0.062 (0.125)	0.032 (0.250)	1 (1)
*Alternaria alternata*	0.062 (0.125)	0.062 (0.062)	1 (8)
*Botrytis cinerea*	0.062 0.250)	0.062 (0.500)	0.062 (8)
*Epicoccum purpurascens*	0.016 (0.125)	0.016 (0.032)	2 (8)
*Cladosporium cladosporioides*	0.016 (0.062)	0.016 (0.500)	0.125 (8)
*Phoma strasseri*	0.016 (0.062)	0.016 (0.032)	0.016 (0.25)
*Pythium debaryanum*	0.062 (0.125)	0.016 (0.032)	1 (1)

**Table 4:** Activity spectrum of EOs.

		A (%) *	
MIC (µL/mL)	Common Thyme	Greek Oregano	Common Oregano
0.016	30	40	10
0.032	30	50	10
0.062	70	70	20
0.125	80	90	30
0.25	100	100	30
0.5	100	100	30
1	100	100	70
2	100	100	80
4	100	100	100
8	100	100	100
16	100	100	100
32	100	100	100
64	100	100	100

A (%) * percentage of microorganisms inhibited by the EOs.

**Table 5:** Climatic parameters.

Month	Year	Min. Temperature (°C)	Max. Temperature (°C)	Rainfall (mm)	Sun Days
January	2017	−7	−2	22.2	19
	2018	−2	2	40.1	18
February	2017	−4	1	35.9	17
	2018	−4	−1	11.8	25
March	2017	2	9	54.0	21
	2018	−3	4	23.2	25
April	2017	3	11	70.7	20
	2018	8	19	19.3	25
May	2017	8	19	73.5	24
	2018	12	23	52.1	23
June	2017	13	23	86.9	20
	2018	13	24	43.6	22
July	2017	14	24	78.5	18
	2018	16	26	120.2	15
August	2017	15	25	64.1	22
	2018	16	27	48.5	22
September	2017	11	18	143.3	20
	2018	12	22	58.8	27
October	2017	7	13	95.6	18
	2018	8	16	53.8	24
November	2017	3	7	53.3	21
	2018	3	8	14.3	27
December	2017	0	4	46.9	19
	2018	−1	3	66.4	20

**Table 6:** Soil parameters.

pH	NO_3_^−^ (mg/L)	NH_4_^+^ (mg/L)	P_2_O_5_ (mg/100 g)	K_2_O (mg/100 g)	Mg (mg/100 g)	Organic Matter (%)
6.25	73	25	23.9	98.0	21.3	2.97

## Data Availability

Data are contained within the article.
